# Larval exposure to insecticides increases adult susceptibility to bluetongue virus in *Culicoides sonorensis* Wirth and Jones (Diptera: Ceratopogonidae)

**DOI:** 10.1007/s10646-026-03183-9

**Published:** 2026-11-10

**Authors:** Blythe E. Bunkers, Bethany L. McGregor, Barbara S. Drolet, Emily G. McDermott

**Affiliations:** 1 Department of Entomology and Plant Pathology, University of Arkansas, Fayetteville, AR 72701 USA; 2 Arthropod-Borne Animal Diseases Research Unit, USDA-ARS, Manhattan, KS USA

**Keywords:** Vector control, Ceratopogonidae, Sublethal, Insecticide, Orbivirus, Bluetongue virus, Insect immunity

## Abstract

*Culicoides* biting midges develop in organically enriched habitats, such as livestock wastewater, which can accumulate pesticide residues from livestock treatments. In some instances, larval development sites are directly targeted with insecticides for vector control. These environmental stressors can affect larval survival and gene expression patterns that govern development and long-term fitness, such as altered immune function. This overlap in gene pathways reflects the integration of detoxification in immunity as a response to both chemical stress and pathogenic threats. As a result, detoxifying pesticides at sublethal levels may developmentally compromise insect vectors, making them more vulnerable to infections in later life stages. In this study, larvae of *Culicoides sonorensis* Wirth and Jones, a major North American bluetongue virus (BTV) vector, were exposed to the lethal concentration for 10% of the test population (LC₁₀) of three distinct classes of insecticides (permethrin, yeast microencapsulated (YME) fluralaner, or methoprene) while controls received equal amounts of solvent (ethanol or water). Survivors were reared to adulthood and offered defibrinated sheep blood spiked with BTV at three days post-emergence. Engorged females were held for up to 14 days post-infection. At 1, 3, 7, 10, and 14 days post-infection, up to 10 females per treatment group were sampled to test for BTV infection and dissemination using RT-qPCR. Infection and dissemination rates, as well as viral titers as quantified by qPCR, were compared across time points and treatments to evaluate whether sublethal pesticide exposure of larvae alters vector competence of adults for BTV. Exposure to three different pesticides resulted in significantly increased BTV titers, particularly at 7 days post-infection (YME fluralaner and methoprene) and 14 days post-infection (permethrin). Infection and dissemination rates at 7 days post-infection were also significantly higher in midges exposed to YME fluralaner and methoprene. Midges exposed to permethrin had significantly higher dissemination rates at 14 days post-infection. Implications of changes in infection dynamics are discussed.

**Supplementary Information:**

The online version contains supplementary material available at https://doi.org/10.1007/s10646-026-03183-9.

## Background

*Culicoides* biting midges (Diptera: Ceratopogonidae) are blood-feeding flies that transmit multiple viral and parasitic agents to animal hosts globally (Kampen and Werner 2023; Mellor et al. 2000; McGregor et al. 2022; Purse et al. 2015; Sick et al. 2019). *Culicoides* are the principal vectors of bluetongue virus (BTV) which is considered one of the most economically important arthropod-borne animal pathogens, with an estimated annual global impact of $3 billion (MacLachlan et al. 1990, McGregor et al. 2022; Mellor et al. 2000; Purse et al. 2015). Biting midges often develop in organically-enriched habitats associated with agricultural production, such as muddy ponds, manure, or wastewater produced by farms (Kampen and Werner 2023; MacLachlan et al. 1990, Mellor et al. 2000; Sick et al. 2019; Barbera et al. 2025). In livestock production environments, numerous chemical pesticides may be applied to animals to control for arthropod pests, and unintended off-target exposure can occur. Larval development sites are prone to collect chemical run-offs, such as herbicides and pesticides used to treat animals (cattle pour-ons, etc.) (Wu et al. 2024).

Pyrethroids are synthetic analogs of pyrethrin, which is derived from chrysanthemum flowers, and are highly effective against a wide range of insect pests, including *Culicoides* (Das 2013; James and Xu 2012; Stenersen 2004). Pyrethroids (e.g. deltamethrin, cypermethrin, flumethrin, permethrin) can be applied as spatial sprays, residual sprays, or as pour-on treatments for animals (Das 2013; Gassner et al. 1997; James and Xu 2012; Liu et al. 2024). And while these treatments are applied directly to the animal, the process of washing and sterilizing the belly and utters of dairy cattle in milking parlors leads to the deposition of chemical residuals into wastewater reservoirs. Dairy wastewater reservoirs are key larval development sites for *Culicoides*, particularly *Culicoides sonorensis* Wirth and Jones, the principal vector of BTV in the United States (US) (Harrup et al. 2016; Mayo et al. 2014). Similarly, insect growth regulators (IGRs) such as methoprene, a juvenile hormone analog, can be administered in several ways in a dairy environment. This includes use as a systemic feedthrough that results in manure that prevents the pupation of larval flies (e.g., face fly, horn fly) (Bruinenberg et al. 2023; Das 2013; Stenersen 2004; Wu et al. 2024; Zhang et al.). Through normal cattle production practices, this waste is washed into wastewater basins, bringing residual methoprene with it (Gassner et al. 1997; Strong 1992). IGRs may also be applied to specific larval development sites to target biting midge juveniles (Palli 2009). And while the development of pesticide resistance is often of concern, the effects of sublethal exposure to pesticides in livestock production on insects’ vector competence are largely unknown.

While innate immune pathways are predominately determined by genotype, phenotypic immune response is influenced by variable extrinsic factors. Stress signal pathways, such as those induced for the detoxification of pesticides, have the ability to jointly influence the immune cascade network (Rosche et al. 2021). Environmental stressors, such as pesticides, can developmentally weaken arthropods when encountered as larvae, through physical damage to the midgut as well as when resources are shunted towards detoxification mechanisms and away from immune pathways in attempt to survive exposure (Aldridge et al. 2024; Angleró-Rodriguez et al. 2017, James and Xu 2012; Mack and Attardo 2023). Sublethal pesticide exposure has been shown to enhance viral dissemination in *Aedes aegypti* L. mosquitoes, affecting their competence for pathogens such as dengue and Zika virus (Aldridge et al. 2024; Moltini-Conclois et al. 2018; Muturi et al. 2011). Yet there are no studies assessing changes in vector competence in *Culicoides* following larval exposure to pesticides.

*Culicoides* found near animal agriculture are likely exposed to insecticides from different classes, particularly pyrethroids and IGRs. Isoxazolines, a newer class of compounds, are increasingly being used in livestock production, and may represent a significant source of insecticide exposure in the future (da Costa et al. 2023). As detoxification pathways and the exerted metabolic effort for successful elimination vary between these insecticidal classes, we hypothesized that vector competence of adult *Culicoides* would vary following larval exposure to any of these three chemical classes versus unexposed controls. To determine whether any class of compounds present a higher risk of adverse downstream effects on BTV competence by *C. sonorensis*, the proportion of midges with midgut infections and disseminated BTV infections in other body tissues, and their respective viral titers, were compared between midges sublethally exposed to each insecticide and unexposed controls. Understanding the effects of sublethal pesticide exposure on vector competence allows for well-informed control tactics and disease management programs to be developed for sustainable agriculture.

## Methods

### Colony maintenance

The *C. sonorensis* Van Ryn (VR) colony, originally established from a dairy in southern California in 1995, was used in this study (Jones et al. 1969). Eggs were provided by the United States Department of Agriculture, Agricultural Research Service (USDA-ARS) in Manhattan, KS, and midges were reared from the egg filter papers at the University of Arkansas. *Culicoides* were maintained at 26 °C and 70–80% relative humidity with a 12:12 photoperiod (Jones et al. 1969). Larval pans (30.5 × 25.5 × 9.5 cm) were made with 1 L of dechlorinated water, two strips of 2.5 × 2.5 × 8 cm polyester quilt batting substrate, 1 mL of nutrient broth (Himedia^®^, MiMedia Laboratories, Maharashtra, India), and 3 g of a mixture of ground alfalfa, powdered livestock protein, egg albumin, and brain heart infusion (“K-medium”; 100:50:1:1) added on the day of pan initiation (Jones et al. 1969). An additional 1 mL of nutrient broth was added to each pan daily. Larval pans were initiated with eggs sheets of the same density (approximately 1200 eggs), to ensure equal access to larval resources prior to pesticide exposure.

### Pesticide preparation

Yeast microencapsulated (YME) fluralaner (Cat. No. B0084-476691, BOCSCI Inc., Shirley, NY) was prepared as described in Lawson and McDermott (2024), and diluted in ddH_2_O to the desired range of concentrations for dose response assays and trials (Lawson and McDermott 2024; US Provisional Patent 63/489,859). S-methoprene (CAS-No: 40596-69-8, Pestanal ®, Sigma-Aldrich, St. Louis, MO) and permethrin (CAS-No: 52645-53-1, Pestanal ®, Sigma-Aldrich, St. Louis, MO) were both dissolved in pure, molecular grade ethanol. Dilutions were made to reach desired concentrations for dose response assays and exposure trials.

### Concentration-response Assays

Preliminary concentration-response trials were conducted to estimate the appropriate lethal concentration resulting in the successful emergence of 90% (LC_10_) of midges following larval exposure to permethrin, YME fluralaner, or methoprene. Dilutions ranged from 1 × 10^− 7^ to 1 × 10^− 3^ mg/mL for permethrin, 1 × 10^− 8^ to 1 × 10^− 4^ mg/mL for YME fluralaner, and 1 × 10^− 9^ to 1 × 10^− 7^ mg/mL for methoprene. Six dilutions were used per chemical, with five replicates per concentration. Permethrin and methoprene were diluted in ethanol, while YME fluralaner was diluted in ddH_2_O, therefore, control groups received an equivalent volume of either ethanol or ddH_2_O, respectively. Deep petri dishes (26 × 100 mm) were prepared with 47 mL of ddH_2_O, 2 × 2 cm polyester batting, 1 mL of nutrient broth, and 10 mg of K-medium. One milliliter of each chemical dilution was added to each replicate prior to adding larvae. For all replicates within all trials, 10 late-stage larvae (L4 for permethrin and YME fluralaner, L3 for methoprene) in 1 mL of water from transfer, were immediately added to each replicate, bringing the total volume to 50 mL. An earlier instar (L3) was used in methoprene trials to reflect the mode of action as a juvenile hormone analog, preventing successful development from the larval to pupal stage. Pupation, emergence, and mortality were observed at 10 days for L4 larvae and 14 days for L3 larvae. Concentration-response assays were conducted independently for each insecticide, and each experiment was repeated twice. A probit analysis (Burgess et al. 2020a, b) was done to then predict the LC_10_ for each chemical.

### Exposure trials

*Culicoides* larvae were exposed to the predetermined LC_10_ of each chemical. For each trial there were two treatment groups: a chemical group and a control group using the corresponding diluent. Trials were repeated three times per experiment. 300–500 larvae per treatment group (L4 for permethrin and YME fluralaner, L3 for methoprene) were counted, removed from colony rearing pans, and transferred to new larval pans within an ACL-2 containment lab by pipette. Experimental pans were prepared in the same manner as colony rearing pans (described above), with the same relative larval density as in the concentration-response assays, and held at 26 °C, 70–80% relative humidity, with a 12:12 light: dark photoperiod. Immediately after larvae were added to the pans, the pans received larvicide treatments in a volume to reach the desired LC_10_. Permethrin treated pans received 14.2 µL from a 0.001 mg/mL stock diluted in 100% ethanol to bring the pan concentration to 1.42e-8 mg/mL. The control pans then received 14.2 µL of 100% ethanol. Similarly, the YME fluralaner pans received 24.9 µL from a 0.000001 mg/mL stock diluted in water to bring the concentration to 2.49e-11 mg/mL, with their control pans receiving an equal amount of water. Methoprene treated pans were treated with 208 µl from a 0.0001 mg/mL stock dissolved in 100% ethanol, to equal 2.08e-9 mg/mL and their controls received 208 µL of 100% ethanol. Pans were mixed thoroughly with a transfer pipette after adding insecticides. Pupae were counted and collected from pans daily, over 7–10 days, into 60 × 15 mm petri dishes fitted with damp cotton, for each treatment group. Pupae dishes were stored at 4 °C prior to being set up to emerge in separate 500 mL cardboard cups with mesh lids. Emerged adults were offered 10% sucrose *ad libitum* via a soaked cotton ball. At 72 h post-eclosion, an infectious blood meal was offered to both control and chemical groups.

Aliquots of BTV-17 (isolate 514664-179; NVSL, Ames, IA, USA) were produced at USDA-ARS Arthropod-Borne Animal Disease Research Unit, Manhattan KS, using 490 cm^2^ roller bottles with 80–85% confluent monolayers of baby hamster kidney cells (BHK − 21; ATCC CCL-10) at MOI = 0.1. Cultures were maintained at 37 °C and 5% CO_2_ in Eagle’s MEM with Earle’s salts, 2% fetal bovine serum, and penicillin/streptomycin sulfate (100 U) until 100% CPE was observed. Virus was harvested by two freeze-thaw cycles followed by centrifugation at 1500 × g for 30 min at 4 °C to pellet cell debris. The titer of the viral stock supernatant was determined by standard plaque assay on Vero MARU cells (VM; Middle America Research Unit, Panama) grown in 199E media containing 2% FBS, 100 µg/mL of streptomycin, 100 units/mL penicillin, and 0.25 µg/mL of amphotericin B at 37 °C with 5% CO_2_. Viral stocks were aliquoted (1 mL) and stored at − 80 °C until shipped on dry ice to the University of Arkansas.

A 750 µL mixture of defibrinated sheep blood (Lampire Biological Laboratories, Pipersville, PA) and BTV (8.5 × 10^6^ PFU/mL) (1:1) was added to 14 mm glass bell feeders (Chemglass Life Sciences, Vineland, NJ) connected to a recirculating water bath set to 37 °C with a Parafilm membrane and positioned on top of the holding containers’ mesh lids. Midges were allowed to feed for 30 min. Both insecticide-exposed and control midges were fed the same infectious blood meal using separate blood feeders. Prior to feeding, an aliquot of the infectious blood meal from each trial was stored at -80 °C for viral RNA quantification using RT-qPCR. After feeding, midges were immobilized with carbon dioxide gas, before being visually assessed and sorted on a chill table under a dissecting microscope. Males and unengorged females (those without visible blood in the abdomen) were removed, while engorged females were held for up to 14 days at 25 °C, 70–80% relative humidity, with a 12:12 light: dark photoperiod with 10% sucrose *ad libitum* in a new 500 mL cardboard cup. At 1, 3, 7, 10, and 14 days post infection (dpi), up to 10 females from each treatment group were removed by aspiration into a 15 mL tube and freezing at -20 °C for 24 h. Once dead, they were removed and placed into a petri dish with 70% ethanol for sorting. Each individual midge was transferred to a 1.5 mL Eppendorf tube filled with 100 µL of RNAlater (Thermo Fisher Scientific, Waltham, MA) and stored at -80 °C for future processing.

### RT-qPCR testing

Individual midges were removed from the RNAlater and placed in a petri dish with 70% ethanol for dissection. The heads were separated from the thorax by blunt dissection using forceps. Both portions of the midge were placed in separate 1.5 mL Safe-Lock tubes with 0.1 mL autoclaved stainless-steel beads (0.9–2.0 mm) (Next Advance Inc., Troy, NY) before adding 200 µL of lysis concentrate buffer solution (Thermo Fisher Scientific, Waltham, MA). All samples were homogenized at speed 8 for 3 min in a Bullet Blender STORM Pro tissue homogenizer (Next Advance Inc., Troy, NY). Samples were held at -80 °C until RNA extraction.

The MagMax™ 96 Viral RNA Isolation Kit (Thermo Fisher Scientific, Waltham, MA) was used to extract viral RNA from homogenized samples using a Kingfisher Flex automated DNA/RNA extraction system (Thermo Fisher Scientific, Waltham, MA). For controls, RNA was similarly extracted from BTV-17 stocks (8.5 × 10^6^ PFU/mL) and serially diluted in ddH2O from [1:1] to [1:10,000]. Extracted RNA was quantified in three technical replicates per sample via RT-qPCR and run in 20 µL reactions by combining 5 µL of extracted sample RNA with 5 µL of Taqman Fast 1-Step Master Mix 4x (Thermo Fisher Scientific, Waltham, MA), 7.8 µL of molecular grade water, 0.5 µM of BTV-NS3-183 F forward primer, 0.5 µM of BTV-NS3-288R reverse primer, and 0.1 µM FAM-labeled BTV-NS3-242 probe (Wernike et al. 2015). Reactions were run on a QuantStudio3 Real-Time PCR System (Applied Biosystems, Waltham, MA) with the following parameters: 50 °C for 5 min, 95 °C for 20 s, and 40 cycles of alternations between 95 °C for 3 s and 60 °C for 30 s. ddH2O was included in each plate as a negative, non-template control. The standard dilution series of BTV-17 stocks (above) were included in each plate as a positive control and to quantify the virus in midge samples. Cycle threshold (Ct) values of < 35 were considered positive (Merrill et al. 2019; Uribasterra et al. 2020).

### Statistical analysis

Data were analyzed using R v4.1.3 and for all analyses, *P* < 0.05 was considered to be statistically significant. A generalized linear model (GLM) was used to determine if a significant interaction between trial and treatment was present prior to pooling data from multiple trials for analysis. The LC_10_ was estimated using probit analyses for each of the three chemicals (Burgess et al. 2020a, b). Model fit was assessed using a chi-square test, and a heterogeneity factor was incorporated if the model failed the goodness of fit test (*P* > 0.05). Abbott’s correction was used to correct for control mortality exceeding 5% (Abbott 1925). Applied Biosystems Design and Analysis software v1.5.1 was used to estimate BTV titers for each positive sample based on that plate’s standard positive dilution series. Viral titer outputs were then converted to Log_10_ values for further analysis. Because midges should not have disseminated infections without also having midgut infections (Fu et al. 2014, Guimerà Busquets et al. 2023), any BTV-positive heads from corresponding BTV-negative bodies were assumed to be contamination and were not included in analyses. BTV-positive head and body proportions (as determined by a Ct < 35) were compared for significant differences using Fisher’s exact test. The average log_10_ BTV titer [PFU/mL] data were assessed for differences in group means and were subject to Shapiro testing to assess normality. Normal data were examined with a Welch t-test to assess for significant differences in group means per time point. Non-normal data were examined with a Kruskal-Wallis test to assess for significant differences in group means.

## Results

### Concentration response assays

#### *Pyrethroid*: permethrin

There was no significant interaction between trial and treatment on mortality, so permethrin trials 1 and 2 were combined for analysis (*P* = 0.30). Average emergence rates ranged from 56.00 ± 36.6% after exposure to 1 × 10^− 7^ mg/mL permethrin (the lowest concentration) to 0.00 ± 0.00% after exposure to 1 × 10^− 3^ mg/mL (the highest concentration). The estimated LC_10_ value for emergence after exposure was 1.4 × 10^− 8^ mg/mL (Table 1).

#### *Isoxazoline*: fluralaner

Fluralaner trials 1 and 2 were combined for analysis (*P* = 0.50). Average emergence rates ranged from 23.00 ± 19.50% after exposure to 1 × 10^− 8^ mg/mL YME fluralaner (the lowest concentration) to 0.00 ± 0.00% after exposure to 1 × 10^− 4^ mg/mL (the highest concentration). The estimated LC_10_ value for emergence after exposure was 2.5 × 10^− 11^ mg/mL (Table 1).

#### *Insect growth regulator*: methoprene

Methoprene trials 1 and 2 were combined for analysis (*P* = 0.20). An average of 88.00 ± 14.00% emergence were observed after exposure to 1 × 10^− 9^ mg/mL methoprene (the lowest concentration) to 63.00 ± 26.70% after exposure to 1 × 10^− 7^ mg/mL (the highest concentration). The estimated LC_10_ value for emergence after exposure was 2.08 × 10^− 9^ mg/mL (Table 1).

**Table 1:** The estimated LC_10_ values (concentration resulting in 10% of exposed larvae failing to successfully emerge as adults) of larval *Culicoides sonorensis* exposed to each insecticide tested

Chemical	*n*	Slope (SD)	LC_10_ mg/mL (95% CI)	X^2^ (df)	*P*-value
Permethrin	500	0.90 (0.09)	1.42e-08 (3.95e-07, 0.002)	25 (2)	3.62e-06
Fluralaner	500	0.59 (0.08)	2.49e-11 (2.59e-08, 3.97e-06)	40 (2)	3.62e-06
Methoprene	500	0.43 (0.08)	2.08e-09 (2.15e-08, 13.7e05)	11 (3)	9.45e-03

### BTV infection assays

**Fig. 1:**
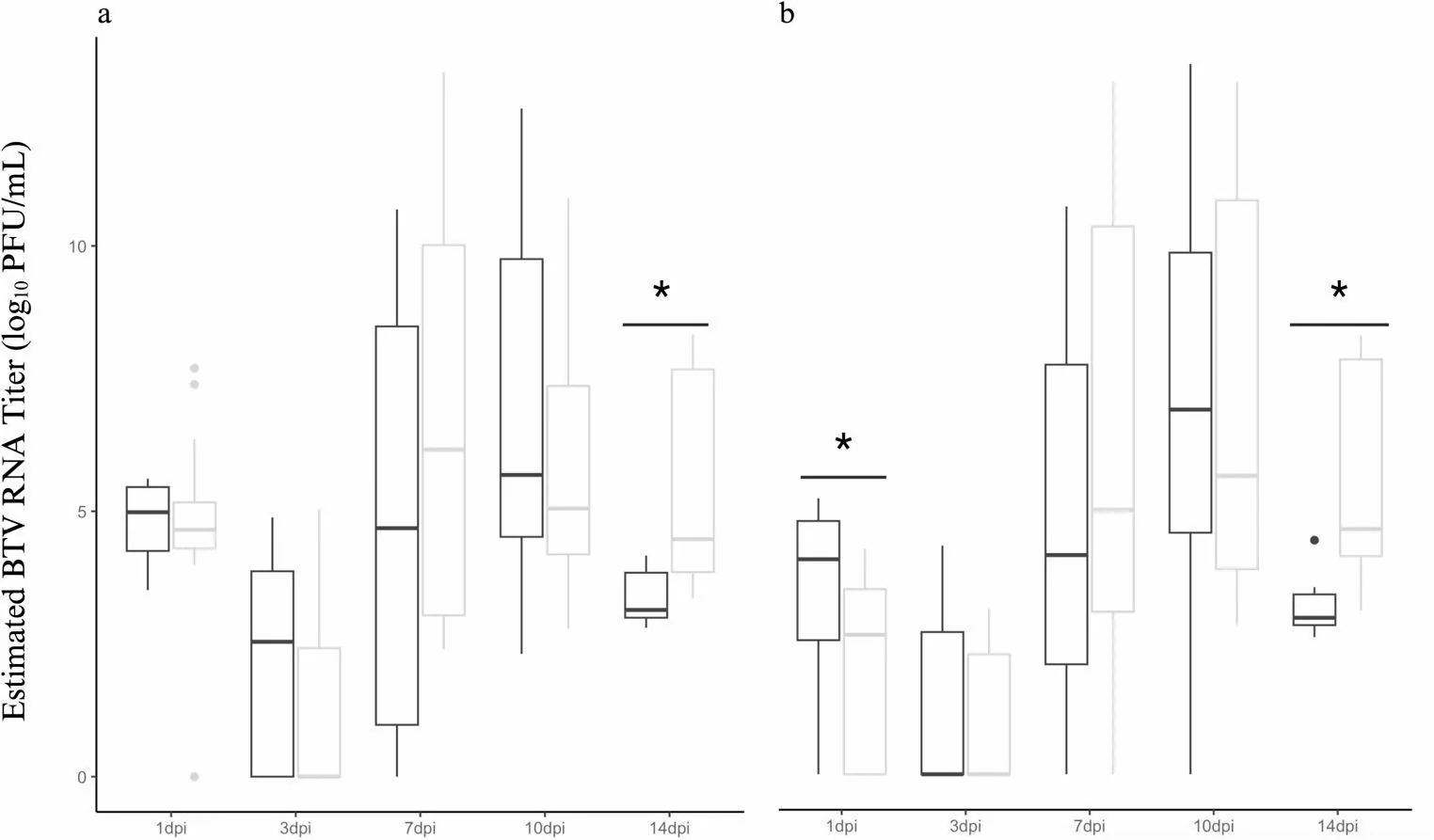
Boxplot depicting the mean body **a** and head **b** BTV titers **[**log_10_(PFU/mL)] for adult *Culicoides sonorensis* after larval exposure to the lethal concentration (LC)-10 of permethrin (grey) (1.42e-08 mg/mL) when compared to the control (black). Dots above/below upper/lower quartiles represent outliers. Asterisks denote significant differences (*P < 0.05, **P < 0.001) between treatments at each time point

Permethrin trials 1, 2, and 3 were pooled for analysis (*P* = 0.25). At 14 dpi, permethrin-exposed midges had significantly higher BTV dissemination rates than control midges, as evidenced by a higher proportion of BTV+ heads (*P* = 0.038) (Table 2). Permethrin-exposed midges also had significantly higher average body (*P* = 0.009, X^2^ = 6.88, df = 1) and head (*P* = 0.005, X^2^ = 7.89, df = 1 ) titers than control midges at 14 dpi (Fig. 1). At 1 dpi, permethrin-exposed midges had a significantly higher head titer than the controls (*P* = 0.013, X^2^ = 6.13, df = 1). There were no other differences in infection or dissemination rates, or in body or head titer at any other time points.

**Table 2:** The number (and percentage) of adult *Culicoides sonorensis* heads and bodies positive for BTV (Ct < 35) at 1, 3, 7, 10 and 14 days post infection (dpi) after larval exposure to the lethal concentration (LC)-10 of permethrin (1.42e-08 mg/mL). Asterisks denote significant differences (*P < 0.05) between treatments at each time point

Time	Treatment	*N*	BTV+ Bodies (%)	BTV+ Heads (%)
1 dpi	LC_10_	24	23 (95.8)	2 (8.33)
	Control	19	19 (100)	5 (26.3)
3 dpi	LC_10_	19	4 (21.1)	0 (0)
	Control	9	3 (33.3)	1 (11.1)
7 dpi	LC_10_	24	17 (70.8)	14 (58.3)
	Control	15	10 (66.6)	8 (53.3)
10 dpi	LC_10_	17	12 (70.6)	11 (64.7)
	Control	14	10 (71.4)	9 (64.3)
14 dpi	LC_10_	12	11 (91.7)	7 (58.3)*
	Control	6	3 (50.0)	0 (0)

**Fig. 2:**
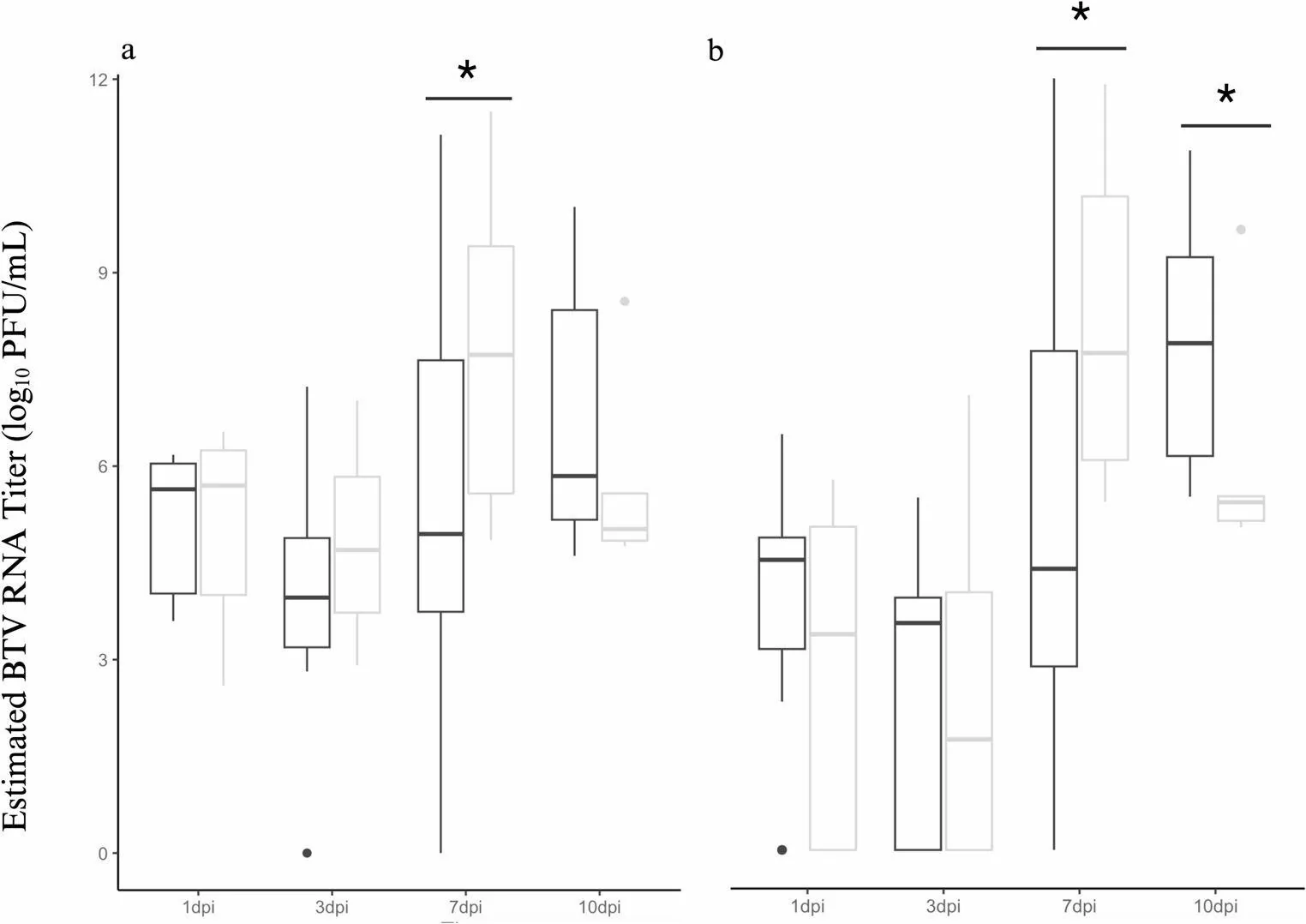
A boxplot depicting the mean body **a **and head **b **BTV titers **[**log_10_(PFU/mL)] for adult *C. sonorensis* after larval exposure to the (LC)-10 of yeast microencapsulated fluralaner (grey) (2.49e-11 mg/mL) when compared to the control (black). Dots above/below upper/lower quartiles represent outliers. Asterisks denote significant differences (*P < 0.05) between treatments at each time point

Fluralaner trials 1, 2, and 3 were pooled for analysis (*P* = 0.37). At 7 dpi, YME fluralaner-exposed midges had significantly higher BTV infection and dissemination rates than control midges, as evidenced by a higher proportion of BTV+ heads (*P* = 0.002) and bodies (*P* = 0.019) (Table 3). Fluralaner-exposed midges also had significantly higher average body (*P* = 0.045, t = -2.10, df = 27.15) and head (*P* = 0.019, t = -2.50, df = 25.04) titers than control midges at 7 dpi (Fig. 2). Head titers in the treatment group dropped to be significantly lower at 10 dpi when compared to control (*P* = 0.047,, X^2^ = 3.93, df = 1). There were no other differences in infection or dissemination rates, or in body or head titer at any other time points.

**Table 3:** The number (and percentage) of *Culicoides sonorensis* heads and bodies positive for BTV (Ct < 35) at 1, 3, 7, 10 and 14 days post infection (dpi) after larval exposure to the lethal concentration (LC)-10 of fluralaner (2.49e-11 mg/mL). Asterisks denote significant differences (*P < 0.05) between treatments at each time point

Time	Treatment	*N*	BTV+ Bodies (%)	BTV+ Heads (%)
1 dpi	LC_10_	16	11 (68.8)	1 (6.25)
	Control	20	20 (100)	5 (25)
3 dpi	LC_10_	10	5 (50)	1 (10)
	Control	11	6 (54.5)	0 (0)
7 dpi	LC_10_	14	14 (100)*	13 (97.9)*
	Control	16	10 (62.5)	6 (37.5)
10 dpi	LC_10_	5	5 (100)	3 (60)
	Control	11	11 (100)	11 (100)

**Fig. 3:**
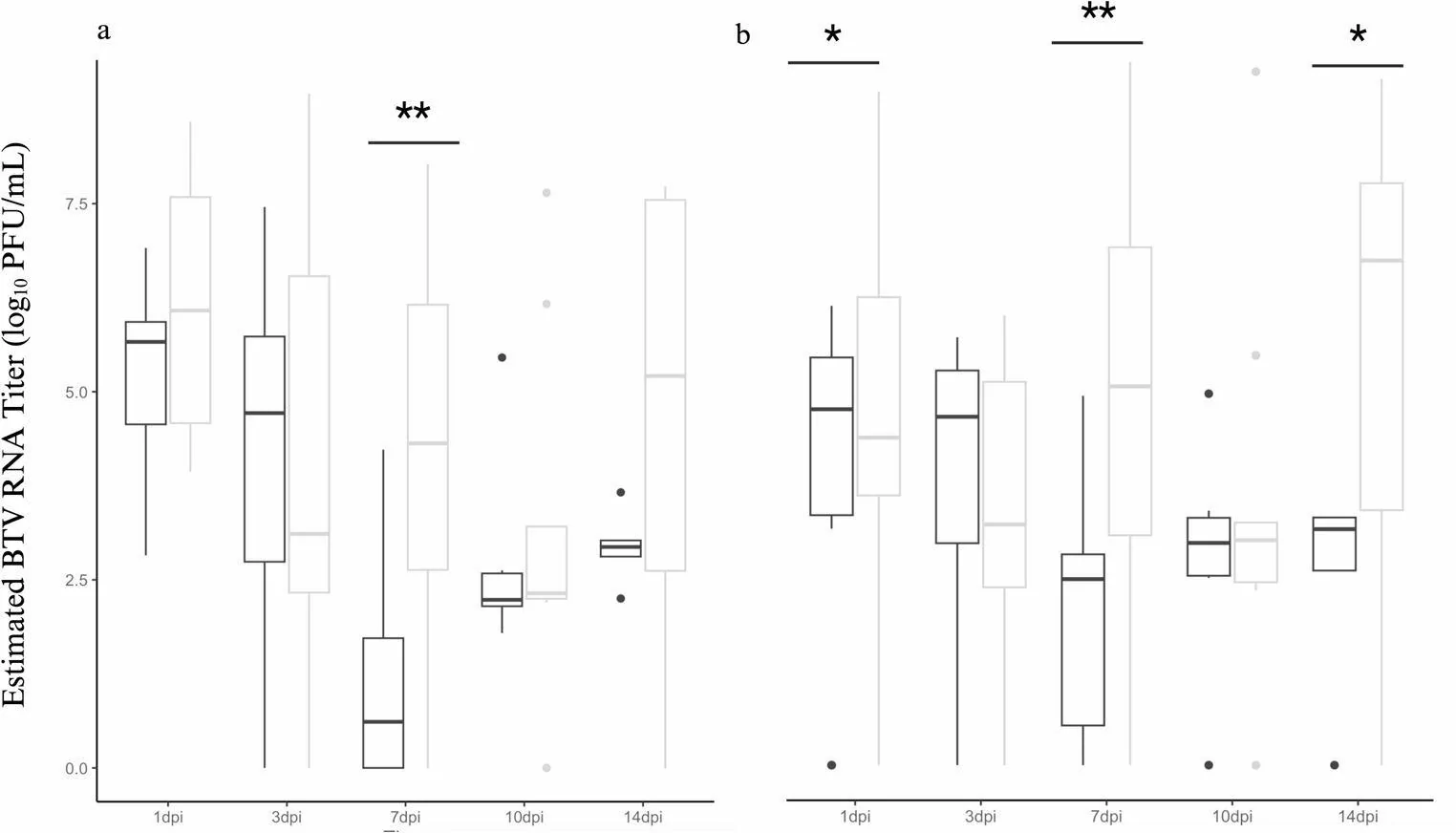
A boxplot depicting the mean body **a** and head **b **BTV titers **[**log_10_(PFU/mL)] for adult *C. sonorensis* after larval exposure to the (LC**)**-10 of methoprene (grey) (2.08e-09 mg/mL) when compared to the control (black). Dots above/below upper/lower quartiles represent outliers. Asterisks denote significant differences (*P < 0.05, **P < 0.001) between treatments at each time point

Methoprene trials 1, 2, and 3 were pooled for analysis (*P* = 0.67). At 7 dpi, methoprene-exposed midges had significantly higher BTV infection and dissemination rates than control midges, as evidenced by a higher proportion of BTV+ heads (*P* = 0.041) and bodies (*P* = 0.046) (Table 4). A significant difference in the number of positive heads was seen at 1 dpi (*P* = 0.046). Methoprene-exposed midges also had significantly higher average body (*P* = 0.0002, t = -4.408, df = 24.30) and head (*P* = 0.005, t = -3.12, df = 25.0 ) titers than control midges at 7 dpi (Fig. 3). Head titers remained significantly higher at 14 dpi (*P* = 0.0304,, t = -2.505, df = 10.37). There were no other differences in infection or dissemination rates, or in body or head titer at any other time points.

**Table 4:** The number (and percentage) of adult *Culicoides sonorensis* heads and bodies positive for BTV (Ct < 35) at 1, 3, 7, 10 and 14 days post infection (dpi) after larval exposure to the lethal concentration (LC)-10 of methoprene (2.08e-09 mg/mL). Asterisks denote significant differences (*P* < 0.05) between treatments at each time point

Time	Treatment	*N*	BTV+ Bodies (%)	BTV+ Heads (%)
1 dpi	LC_10_	20	20 (100)	11 (55)*
	Control	15	14 (93.3)	3 (20)
3 dpi	LC_10_	25	6 (24)	5 (20)
	Control	13	4 (30.8)	1 (7.7)
7 dpi	LC_10_	19	12 (63.2)*	11 (57.9)*
	Control	10	1 (10)	1 (10)
10 dpi	LC_10_	10	2 (20)	2 (20)
	Control	7	2 (28.6)	2 (28.6)
14 dpi	LC_10_	8	5 (62.5)	5 (62.5)
	Control	5	2 (40)	0 (0)

## Discussion

Insects exposed to environmental stressors, including insecticides, experience fitness costs (Aldridge et al. 2024; Angleró-Rodriguez et al. 2017, James and Xu 2012; Mack and Attardo 2023). While exposure to high concentrations of insecticides will obviously result in death, insects are sometimes able to detoxify lower concentrations and survive (Andreazza et al. 2021; Kouame et al. 2026; Zeferino et al. 2025). These surviving individuals may be less fit than unexposed insects due to either direct injury or resource reallocation from detoxification. As vector competence in adult mosquitoes is known to be affected by stress in the larval stages, we hypothesized that sublethal insecticide exposures in *Culicoides* larvae might alter their ability to transmit BTV as adults (Aldridge et al. 2024; Muturi et al. 2011; Richards et al. 2017). In an attempt to investigate the interaction between immune response and pesticide detoxification in *C. sonorensis* biting midges, three classes of insecticides were chosen for this study as they have distinct modes of action, chemical structures, and involve different detoxification mechanisms to varying degrees (Das 2013; James and Xu 2012; Stenersen 2004).

*Culicoides sonorensis* exposed to permethrin as larvae exhibited significantly higher mean body and head titers when compared to the control group at 14 dpi. Also at the 14 dpi time point, the treatment group had a significantly higher proportion of BTV positive heads, or disseminated infections. At 1 dpi, a significant difference in head titer between treatment and control is reported, but this is likely due to undigested bloodmeal in the food canal at this time point, and not viral dissemination. Some virus can be detected in *Culicoides* orally infected with BTV at early time points, though titers increase after 1 dpi and infections are not generally transmissible until at least 7 dpi at temperatures ~ 25 °C (Fu et al. 1999; Wittmann et al. 2002). Permethrin exposure led the treatment group to have higher infection and dissemination rates by day 14 when compared to the control, indicating that the LC_10_ permethrin had lasting effects on vector competence. While this may be true, it is unlikely for bloodfed individuals to live 14 days post-feeding (Dreyer and Vaughan 2022; Gerry and Mullens 2000). Midges would most likely have begun, or completed, a second gonotrophic cycle (allowing for transmission) before 14 dpi, when BTV infection between the insecticide-exposed and control groups was equivalent. The increased dissemination rates observed at 14 dpi in the permethrin-exposed group may not be ecologically important. Therefore, while the data show significant differences in titers and proportion positive at day 14, these individuals would not necessarily be considered to present an increased risk of BTV transmission to livestock.

The larval midges exposed to fluralaner during development exhibited increased rates of viral infection and dissemination, but more quickly than those exposed to permethrin. At 7 dpi, there were significantly higher mean body and head titers in the treatment group when compared to the control, as well as significantly higher proportions of BTV positive heads and bodies. Disseminated infections happened at a faster rate, more frequently, and persisted longer in midges exposed to an isoxazoline as larvae. The extrinsic incubation period, or time that it takes for the insect to become infectious to other hosts, was shorter in the treatment group (7 dpi) compared to the controls (10 dpi). As extrinsic incubation period is a factor in vectorial capacity (Brady et al. 2016), this change could increase overall BTV transmission by *C. sonorensis* for BTV, by reducing the period it takes for the virus to replicate, disseminate, and thereby become transmissable. As evidenced by these data, both vector competence and vectorial capacity are directly impacted by exposure to an isoxazoline at a sublethal concentration.

Isoxazolines are a relatively newer insecticide class, and limited detoxification studies have been done on these compounds. Because of its novelty, biting midges may not currently be exposed to fluralaner in most countries, but Exzolt^®^, a fluralaner-based pour-on treatment for cattle, has been recently approved in Brazil and Australia (Berny et al. 2024). In December 2025, Exzolt™ Cattle-CA1 was also conditionally approved by the US Food and Drug Administration in response to the threat of New World screwworm in North America. As the use of topical isoxazolines in livestock is expanded, the implications of sublethal exposure presented here must be critically understood for BTV management among ruminant producers.

The same pattern was observed in the methoprene experiment, where mean BTV titers and the proportion of *Culicoides* with disseminated infections were significantly higher in the insecticide-exposed group at 7 dpi, indicating that these individuals present an increased risk of BTV transmission to livestock. At 1 dpi, a significantly higher proportion of BTV+ heads is reported in the treatment group, but again is likely due virus in the remnant bloodmeal and not true dissemination. As reported here, the increased susceptibility to BTV as adults after exposure to an IGR as larvae, matches previous studies demonstrating increased DENV susceptibility in mosquitoes after IGR exposure. Similar to the fluralaner treatment group, the methoprene exposed insects demonstrated shorter extrinsic incubation times and increased vector competence for BTV.

IGRs (e.g., methoprene, pyriproxyfen, novaluron) are chemicals that interfere with the development of insect larvae, and resistance in mosquitoes is linked to overexpression of P450, resulting in enhanced oxidative degradation of juvenoids (Cornel et al. 2002; Jiang & Burroughs 2025, Palli 2009; Hammock et al. 1977). GST and esterases are also involved in detoxification by hydrolyzing the ester bond in methoprene (Hammock et al. 1977). P450 has been found to be the primary detoxification pathway for fluralaner in *Solenopsis invicta* Buren (Xiong et al. 2020) and *Musca domestica* L. (Norris et al. 2023). Because of the similar change in dissemination rate, we hypothesize that similar detoxification pathways are involved after exposure to isoxazolines and IGRs in biting midges. Detoxification of pyrethroids, such as permethrin, is also predominately achieved by P450 enzymes, and secondarily by carboxylesterases (Gong et al. 2022; Poupardin et al. 2008; Yang et al. 2021). Detoxification of all three insecticide classes used in this study involve the P450 superfamily (Xiong et al. 2020; Norris et al. 2023; Cornel et al. 2002; Jiang & Burroughs 2025, Palli 2009), but seeing that permethrin exposure did not have this same effect, it may be a specific subset of P450 enzymes involved. Furthermore, the detoxification of isoxazolines is understudied, leading to knowledge gaps that may provide insight into this observation.

In mosquitoes, a variety of genes are upregulated in response to exposure to stressors, such as insecticides, plant toxins, or immune challenges (James and Xu 2012; Prince et al. 2023; Sim et al. 2014). The genes governing Toll and immunodeficiency (IMD) pathways are upregulated under conditions of stress and may contribute to cross-protection between immune defense and detoxification mechanisms (James and Xu 2012; Rosche et al. 2021). Alternatively, in *Culicoides* biting midges, immune gene TOLL-7 was downregulated following vesicular stomatitis virus (VSV) infection in vitro (Scroggs et al. 2023) and epizootic hemorrhagic disease virus (EHDV) infection in vivo (Nayduch et al. 2019), yet upregulated following a disseminated BTV infection (Morales-Hojas et al. 2018). There is a potential overlap in how an arthropod might coordinate both detoxification and immune responses to stressors like insecticides and pathogens. For example, the Toll pathway has also been shown to be involved in regulating the expression of detoxification genes (James and Xu 2012; Rosche et al. 2021). Conversely, because of the metabolic prioritization of detoxification for survival, many studies similar to the one at present report increased viral susceptibility, infection rates, and dissemination rates after sublethal pesticide exposure. *Aedes albopictus* Skuse exposed to sublethal bifenthrin (a pyrethroid) as adults had significantly lower dengue virus (DENV) infection rates and significantly higher Zika virus transmission rates (Wang et al. 2023; Richards et al. 2017). Similarly, *Ae. aegypti* exposed to malathion (an organophosphate) as larvae exhibited increased sindbis virus dissemination rates as adults (Muturi et al. 2011). Additionally, larval *Ae. aegypti* exposed to the insect growth hormone analogs, methoprene and pyriproxyfen (IGRs), had significantly increased susceptibility to DENV infection as adults (Aldridge et al. 2024).

Innate immunity plays a crucial role in determining vector competence by influencing the replication of pathogens within the insect (Carpenter et al. 2015; Kramer and Ciota 2015; Mullens et al. 2004; Mills et al. 2017; Nayduch et al. 2014; Sick et al. 2019). Based on our results, we hypothesize that the metabolic burden of detoxification impacts innate immunity in *C. sonorensis*, resulting in increased dissemination of BTV at earlier time points. While detoxification of the chemical pesticides is not actively happening at the time of BTV infection, the stress experienced as larvae may lead to fitness changes overtime (Muturi et al. 2011; Yan et al. 2023). Environmental stressors, such as competition or pesticides, can have profound and lasting effects on larval survival and gene expression patterns that govern development and long-term fitness, such as altered immune function. This overlap reflects the integration of detoxification in immunity as a response to both chemical stress and pathogenic threats. Future studies should include sampling data from larval habitats to accurately assess residual pesticide levels in the environment to better predict true exposure limits. In the experimental design of this study, the LC_10_ was arbitrarily choosen as to allow enough larvae to survive to adulthood for viral infection assays. If accurate environmental concentrations were determined, experimental outcomes may more precisely reflect the impact of sublethal effects in the field. Furthermore, other classes of chemicals could be tested in this manner, as different classes involve different detoxification pathways that would uniquely overlap immune pathways. Finally, exposure trials such as these could be done in conjunction with transcriptomic studies to specifically pinpoint changes in gene regulation modulating immune responses that lead to the phenotypic changes observed here. 

## Supplementary Information

Below is the link to the electronic supplementary material.

Supplementary Material 1

Supplementary Material 2

## Data Availability

Data supporting these conclusions are included within the article.

## Abbreviations

BTV: Bluetongue virus
IGR: insect growth regulator
IMD: immune deficiency pathway
GABA: gamma-aminobutyric acid
PAMP: pathogen associated molecular patterns
PRR: pattern recognition receptor
RNAi: interference ribonucleic acid
AMP: antimicrobial peptide
RT-qPCR: reverse transcriptase quantitative polymerase chain reaction
ANOVA: analysis of variance
GLM: generalized linear model
CYP: cytochrome P450s
GST: glutathione S-transferases
ROS: reactive oxygen species
LC: lethal concentration
YME: yeast microencapsulated
Ct: cycle threshold
