# Supplementary material for: Larval exposure to insecticides increases adult susceptibility to bluetongue virus in Culicoides sonorensis Wirth and Jones (Diptera: Ceratopogonidae)
Source: Ecotoxicology. 2026 Nov 10;35(10):200. doi: 10.1007/s10646-026-03183-9 (PMC13751548; doi:10.1007/s10646-026-03183-9)
Supplement: Supplementary file 1 [file 10646_2026_3183_MOESM1_ESM.pdf]

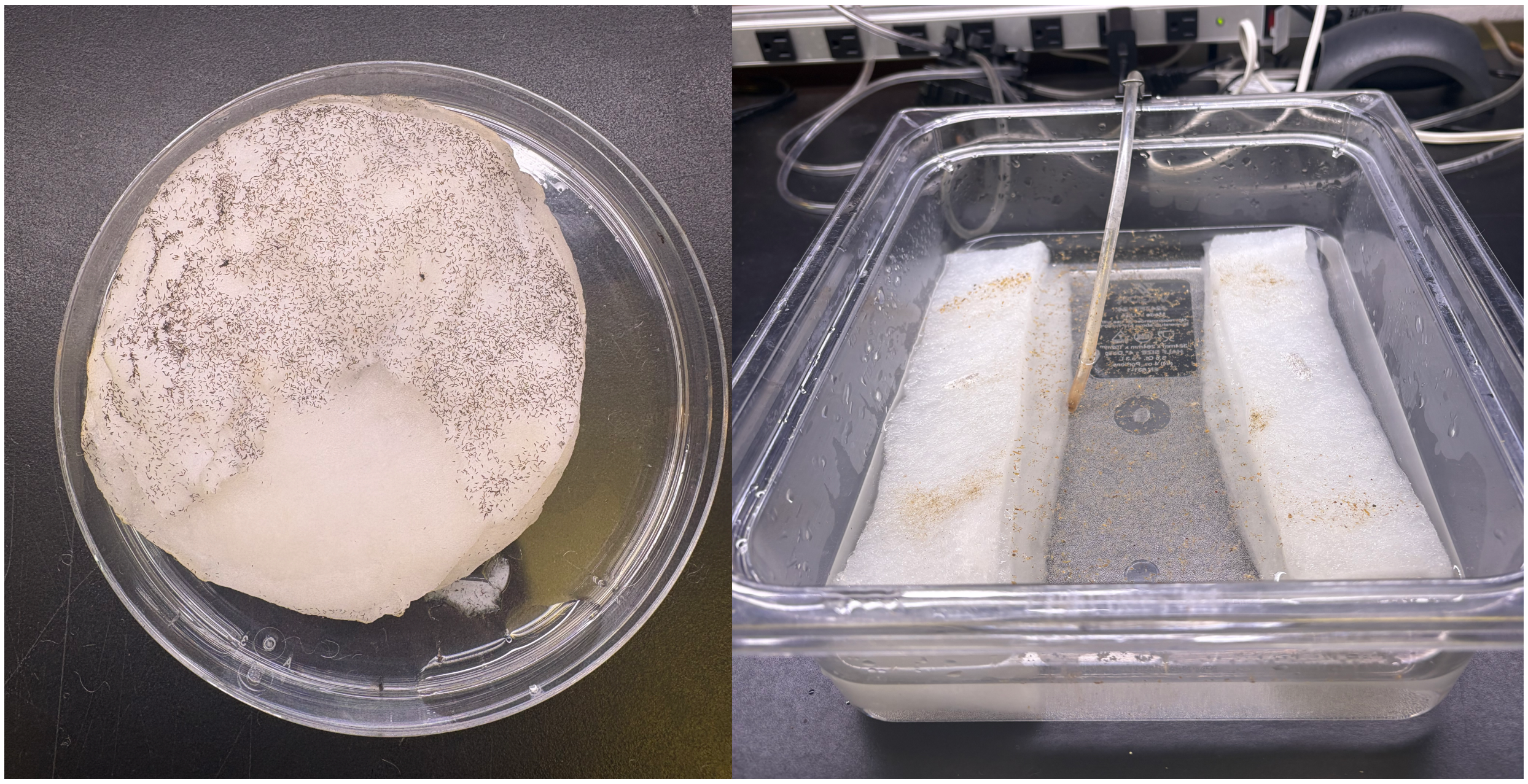

A) **B)**

Supplementary Figure 1. A) an egg sheet and B) a larval pan for *Culicoides sonorensis*.
