# Supplementary material for: Larval exposure to insecticides increases adult susceptibility to bluetongue virus in Culicoides sonorensis Wirth and Jones (Diptera: Ceratopogonidae)
Source: Ecotoxicology. 2026 Nov 10;35(10):200. doi: 10.1007/s10646-026-03183-9 (PMC13751548; doi:10.1007/s10646-026-03183-9)
Supplement: Supplementary file 2 [file 10646_2026_3183_MOESM2_ESM.pdf]

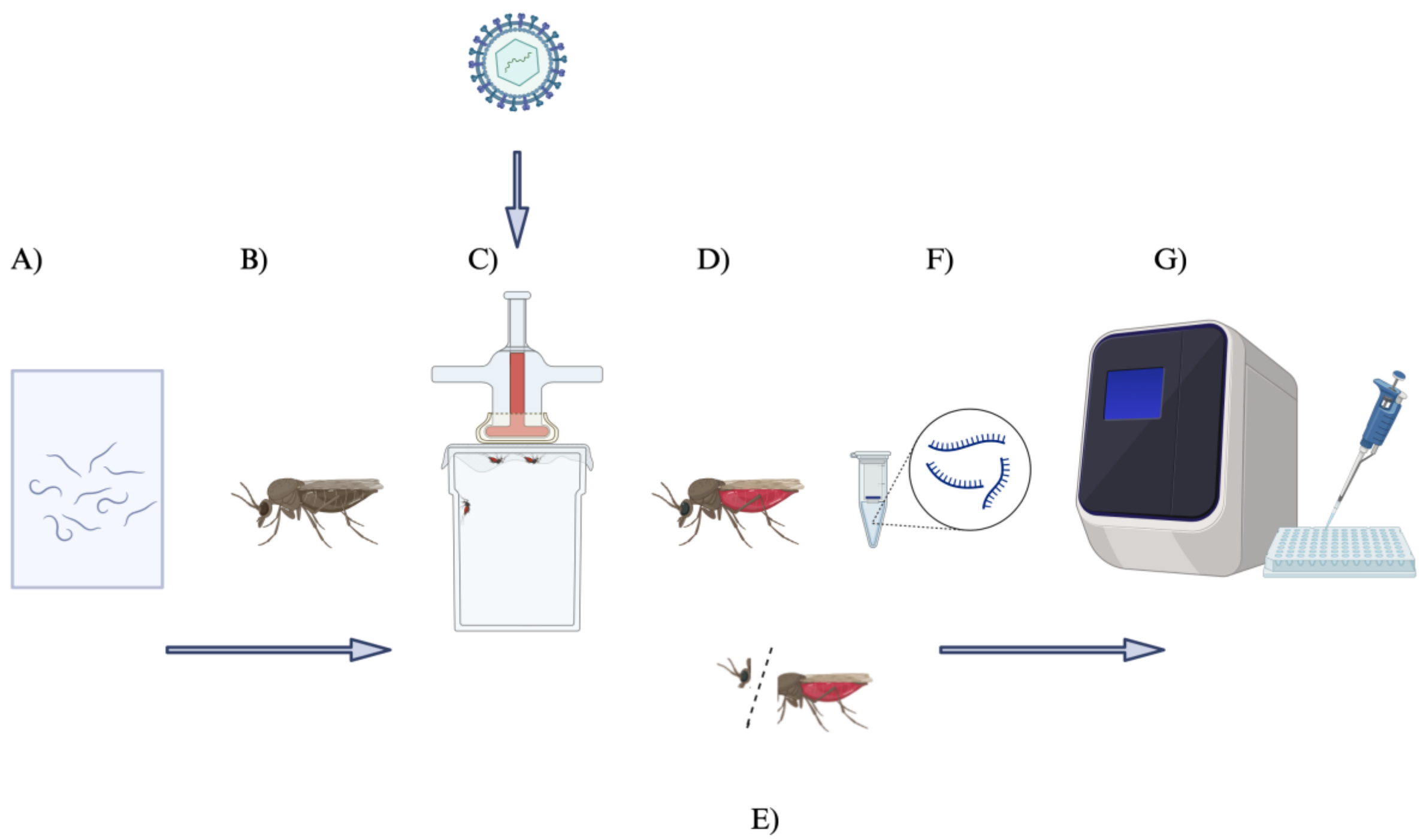

Supplementary Figure 2. A graphical flowchart depicting the experimental design. A) *Culicoides sonorensis* larvae are reared out in pans respective to treatment group, B) adults are sorted by sex and females are set up to blood feed in either the BTV or control group, C) tittered BTV viral stocks are used to spike bloodmeals, D) bloodfed females are held for 1 – 14 days, E) heads are bluntly dissected from bodies for each sample, F) BTV DNA is extracted from the head (dissemination) and body (infection) samples, G) RT-qPCR is used to quantify the viral titers from samples according to the known titers of standards.
